# Does an ‘Activity-Permissive’ Workplace Change Office Workers’ Sitting and Activity Time?

**DOI:** 10.1371/journal.pone.0076723

**Published:** 2013-10-02

**Authors:** Erin Gorman, Maureen C. Ashe, David W. Dunstan, Heather M. Hanson, Ken Madden, Elisabeth A. H. Winkler, Heather A. McKay, Genevieve N. Healy

**Affiliations:** 1 Centre for Hip Health and Mobility, Vancouver, British Columbia, Canada; 2 University of British Columbia Department of Family Practice, Vancouver, British Columbia, Canada; 3 Baker IDI Heart and Diabetes Institute, Melbourne, Victoria, Australia; 4 University of British Columbia Department of Medicine, Vancouver, British Columbia, Canada; 5 University of British Columbia Department of Orthopaedics, Vancouver, British Columbia, Canada; 6 The University of Queensland, School of Population Health, Brisbane, Queensland, Australia; 7 Monash University, Epidemiology and Preventive Medicine, Melbourne, Victoria, Australia; 8 The University of Western Australia, School of Sports Science, Exercise and Health, Perth, Western Australia, Australia; University of Washington, United States of America

## Abstract

**Introduction:**

To describe changes in workplace physical activity, and health-, and work-related outcomes, in workers who transitioned from a conventional to an ‘activity-permissive’ workplace.

**Methods:**

A natural pre-post experiment conducted in Vancouver, Canada in 2011. A convenience sample of office-based workers (n=24, 75% women, mean [SD] age = 34.5 [8.1] years) were examined four months following relocation from a conventional workplace (pre) to a newly-constructed, purpose-built, movement-oriented physical environment (post). Workplace activity- (activPAL3-derived stepping, standing, and sitting time), health- (body composition and fasting cardio-metabolic blood profile), and work- (performance; job satisfaction) related outcomes were measured pre- and post-move and compared using paired t-tests.

**Results:**

Pre-move, on average (mean [SD]) the majority of the day was spent sitting (364 [43.0] mins/8-hr workday), followed by standing (78.2 [32.1] mins/8-hr workday) and stepping (37.7 [15.6] mins/8-hr workday). The transition to the ‘activity-permissive’ workplace resulted in a significant increase in standing time (+18.5, 95% CI: 1.8, 35.2 mins/8-hr workday), likely driven by reduced sitting time (-19.7, 95% CI: -42.1, 2.8 mins/8-hr workday) rather than increased stepping time (+1.2, 95% CI: -6.2, 8.5 mins/8-hr workday). There were no statistically significant differences observed in health- or work-related outcomes.

**Discussion:**

This novel, opportunistic study demonstrated that the broader workplace physical environment can beneficially impact on standing time in office workers. The long-term health and work-related benefits, and the influence of individual, organizational, and social factors on this change, requires further evaluation.

## Introduction

Within the office workplace, prolonged sitting is common while time spent physically active (either standing, in light intensity activity, or in moderate-to-vigorous intensity activity) is minimal [1-4]. There are several drivers for these behaviors including organizational norms, task design, policy, and individual preference [5,6]. One integral element that can promote or restrict activity is the physical environment [6,7]. Traditional building design is biased toward “human energy conservation” with many sitting options available [8]. This is of concern as there is now considerable evidence that excessive sitting, particularly sitting time accumulated in prolonged, unbroken bouts [9-11], is detrimentally associated with several health outcomes including type 2 diabetes, cardiovascular disease, and premature mortality [12,13]. Importantly, these associations are observed even in adults who meet the public health guidelines for physical activity [14,15]. Conversely, regularly interrupting sitting time is beneficially associated with biomarkers of cardiovascular health, including waist circumference, glucose and insulin [9-11]. Even just replacing some sitting with standing can substantially increase leg muscle activity [16], with corresponding health benefits [17].

‘Activity-permissive’ buildings, containing features such as visible, easily accessible, and visually appealing stairs, appealing activity permissible spaces, and amenities such as transit and bike facilities [6,7,18], may beneficially impact employees’ physical activity and sitting time, and subsequently their health and possibly work-related outcomes [19,20]. In children, a move from a traditional classroom to a purpose-built activity-permissive classroom (an enclosed 35,000 feet^2^ space with several activity promoting features including vertical, mobile white-boards; portable video display units; and, standing desks as well as allowance of free movement during lesson plans) showed a significant and substantial increase in movement [21]. In office workers, considerable (>2 hours per 8-hour workday) reductions in workplace sitting time have been observed following the introduction of an individual-level physical environment modification (sit-stand workstation) [4,22]. However, the extent and magnitude of any such benefits for office workers following a whole-of-workplace environmental change (i.e. large, structural changes that are designed to facilitate and encourage movement) are unknown. Although evaluating the consequences of these large-scale workplace interventions is complex, a natural experiment facilitates an ecologically valid perspective of the implication(s) of such changes.

Therefore the objective of this novel natural experiment was to evaluate changes in workplace activity (i.e. standing and stepping time) and sitting time, as well as health- and work-related outcomes, in office-based workers before and after transitioning from a conventional workplace to an ‘activity-permissive’ physical workplace environment. It was hypothesized that pre-post move: sitting time would decrease; standing and stepping time would increase; health outcomes and job satisfaction would improve; and, work performance would be unchanged.

## Methods

### Study design

This study used a pre-post design. Data were collected pre-move (February-June 2011), and an average four months post-move (October-December 2011: range 3-6 months), and were analyzed in December 2012. A minimum of three months occupancy in the new building was chosen to permit workers to acclimatize to the new work environment. The study was approved by the University of British Columbia (UBC) and Vancouver Coastal Health ethics board.

### Participants

All office-based employees (staff, graduate students, faculty) from an academic physical activity research centre moving to the ‘activity-permissive’ building were invited to participate (n=79). Eligibility criteria were: ambulatory, non-pregnant, and ≥0.8 full-time equivalent. All participants provided written informed consent.

### Workplaces

#### Pre-move

The original workplace was two connected retrofitted hospital buildings, converted into office space that was predominantly of closed design with no standing options in offices or meeting rooms.

#### Post-move

**Figure 1:**
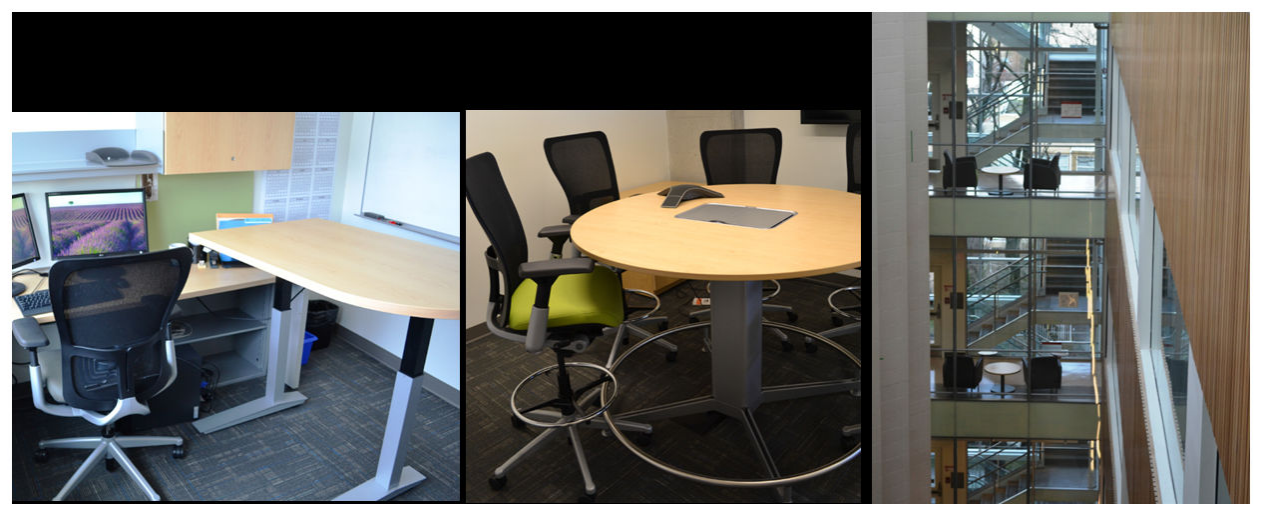
Some of the features of the new ‘activity permissive’ building. Specific features included adjustable standing desks, café-style meeting rooms with options to sit or stand, and glass staircases to maximize light and visibility.

The building for the new workplace was a purpose-built (i.e., specifically designed for the research group moving to the building), activity-permissive physical environment. Building features included an internal glass enclosed staircase (with attractive views) as a main design feature (Figure 1), electric height-adjustable workstations at selected desks (faculty-only), standing-option meeting rooms and common areas, centralized supplies/printing, and an office layout that promoted vertical integration (key destinations traversed different floors). Participants received no information or education regarding the potential benefits of sitting less and moving more at work and/or how the move to the new building could facilitate this. Although participants were from a physical activity research centre, at the time of the study, the potential health impacts of prolonged sedentary behavior was just emerging in the academic literature and mainstream media.

### Data collection

For both pre- and post-move, participants wore an activPAL3 activity monitor continuously (i.e. 24 hours/day) for seven days (PAL Technologies Limited, Glasgow, UK), completed an interviewer-administered questionnaire, and underwent anthropometric and fasting (minimum 8 hours) blood measurement. Participants were requested to refrain from any moderate-to-vigorous intensity exercise, alcohol, or caffeine for 24 hours prior to each blood measurement. At each time-point, participants attended two measurement sessions eight days apart. At the first measurement session, the questionnaires were completed, blood measures were taken, and the activity monitor was attached. At the second measurement session, anthropometric measures were taken and the activity monitor was collected.

### Measures

Activity outcomes were measured by the valid and responsive [23-25] activPAL3 activity monitor (version 6.3.0; default settings). This small (53 x 35 x 7mm; 15g), unobtrusive monitor is a capacitative accelerometer that collects raw triaxial activity data at 20 Hz, and classifies it into periods spent sitting/lying, standing, and stepping (i.e., walking). This activity monitor was waterproofed (with a finger cot and waterproof surgical dressing), secured onto the right anterior thigh with a hypoallergenic patch, and worn 24 hours/day for each 7-day assessment period. Participants recorded via a logbook times spent at the primary workplace, awake/asleep times, and monitor removal (if any).

Using SAS 9.2 (SAS Institute Inc., Cary, North Carolina USA) and activPAL3 event files, activPAL3 bouts that were predominantly (≥50%) worn, awake and at the workplace were used to determine average stepping, standing, and sitting time (total, and time accumulated in bouts ≥30 minutes [prolonged]), and the number of sit-to-stand transitions at the workplace on valid days. For this analysis, days were valid if the activPAL3 was worn for ≥80% of time at the workplace. To account for variations in work time, and to facilitate comparisons to previous research [4,22], these outcomes were standardized to an eight-hour workday [standardized minutes = outcome minutes * 480/observed workplace minutes], except for sit-to-stand transitions which were reported per hour of workplace sitting.

Height and weight was measured using standard procedures. Dual energy X-ray absorptiometry (DXA; Hologic QDR 4500W, Hologic Inc., Bedford, MA) and manufacturer recommended protocols [26] were used to derive whole-body (excluding the head) percent fat mass. Fasting blood samples were taken in the morning on the first day of each study phase. Samples were obtained via venipuncture and were analyzed at the local hospital laboratory (Vancouver General Hospital). Plasma glucose (hexokinase), high-density lipoprotein cholesterol and triglycerides (enzymatic-colorimetric), and *C-reactive protein* (CardioPhase High Sensitivity CRP) were assessed on a Siemens Dimension Vista 1500; serum for insulin assays was frozen at -20 degrees Celsius and measured in a single batch each time period (electrochemiluminescence immunoassay) on a Siemens Advia Centaur.

Data on socio-demographic characteristics were collected pre-move. Self-rated work performance (9-item, 10-point scale [27]) and job satisfaction (“How would you rate your satisfaction with your job?”: 5-point Likert scale: one very unsatisfied; five very satisfied) were collected at both time points, while post-move, participants were asked the extent to which they strongly disagreed (1) to strongly agreed (5) with the statements: “overall, I enjoy the new workplace”, and “the move to the new workplace has improved my productivity”.

### Statistical Analysis

Descriptives are reported as mean [SD]. Changes were examined using paired *t*-tests, reporting mean change [95% CI], with data reported as log-transformed means for *C-reactive protein* (not normally distributed). Given that only faculty had access to the flexible desks, one-way ANOVAs (Tukey post-hoc) were used to evaluate if there were any differences in activity change outcomes by employment level (faculty, staff, graduate student). Analyses were conducted in SPSS Version 21 (IBM Corp); statistical significance was set at p<0.05 (two-sided), with the confidence intervals used to interpret the potential magnitude of the effect [28].

## Results

Twenty-seven (of 79: 34%) employees moving to the new building volunteered for the study and completed pre-move assessments. Three participants left the workplace; the remaining 24 eligible participants (18 women, mean [SD] age = 34.5 [8.1] years) all completed the post-move assessment and were included in the analyses. Participants were highly educated (75% minimum Master’s education). The distribution of faculty (n=4; 16%), staff (n=7; 29%), and graduate students (n=13; 54%) who participated was comparable to broader distribution of those employment types within the workplace (18% faculty, 39% staff, 43% graduate students out of 79). Across all participants, valid workplace data was available for 103 days (out of 104 recorded days) pre-move, and 105 days (out of 105 recorded days) post-move.

Activity, health-, and work-related outcomes are reported in Table 1. Pre-move, most workplace time on average was spent sitting (364 [SD 43.0] mins/8-hr workday or 75.9 [9.0]% of work time), followed by standing (78.2 [32.1] mins/8-hr workday) and stepping (37.7 [15.6] mins/8-hr workday), with much (55%) of the sitting time accumulated in prolonged, unbroken bouts of at least 30 minutes. Post-move, there was a significant increase in workplace standing time [+18.5, 95% CI: +1.8, +35.2 mins/8-hr workday], which was more likely due to a reduction in sitting time (-19.7, 95% CI: -42.1, +2.8 mins/8-hr workday) than changes in stepping time (+1.2, 95% CI: -6.2, +8.5 mins/8-hr workday, respectively). The individual variation of this change was considerable for these outcomes, with change varying by -158 to +74 mins/8-hr workday for sitting; -41 to +129 mins/8-hr workday for standing, and -32 to +46 mins/8-hr workday for stepping. Sitting (F(2, 21)=4.493, p=0.022) and standing (F(2,21)=4.281, p=0.028) change varied significantly by employment level, with post-hoc tests showing staff reduced their sitting time (-60.7 ± 73.6 mins/8-hr workday) and increased their standing time (+48.6 ± 56.0 mins/8-hr workday) significantly more than faculty (sitting: +21.9 ± 37.7 mins/8-hr workday; standing: -11.0 ± 23.5 mins/8-hr workday). There were no statistically significant differences between the graduate student group and the staff or faculty groups for any activity outcome. Sitting time accumulation did not vary substantially pre-post move, with non-significant changes in sitting time accumulated in prolonged, unbroken bouts (+1.9 [-32.4, 36.2] mins/8-hr workday) and in number of sit-to-stand transitions (-0.4 [95% CI -1.01, 0.21]). However, the broad confidence intervals around prolonged sitting time do not rule out meaningful changes (both beneficial and adverse) for this outcome.

**Table 1:** Workplace activity, health, and work-related outcomes before and after transitioning to an activity-permissible workplace (Vancouver, Canada, 2011: n=24).

**Measure**	**Mean (SD) or median (25^th^, 75^th^ percentile)**		**Post - pre intervention**	
	**Pre-intervention**	**Post-intervention**	**Mean Change (95% CI)**	**p**
Observed workplace time, mins	420.2 (90.4)	449.8 (81.0)	29.7 (-6.1, 65.4)	
***Activity monitor outcomes***				
Stepping time, mins/8-hr workday	37.7 (15.6)	38.9 (16.5)	1.2 (-6.2, 8.5)	0.748
Standing time, mins/8-hr workday	78.2 (32.1)	96.7 (41.9)	**18.5 (1.8, 35.2)**	0.032
Sitting time, mins/8-hr workday	364.1 (43.0)	344.4 (53.1)	-19.7 (-42.1, 2.8)	0.084
Time accrued in prolonged sitting ≥30 min, mins/8-hr workday	202.1 (95.5)	204.0 (87.5)	1.9 (-32.4, 36.2)	0.909
Sit-to-stand transitions, n/hr workplace sitting	3.6 (1.7)	3.2 (1.3)	-0.4 (-1.01, 0.21)	0.185
***Health outcomes***				
Weight, kg	67.8 (12)	67.4 (11.7)	-0.34 (-1.1, 0.5)	0.387
Percent Fat, % ^a^	26.6 (8.9)	26.6 (9.4)	-0.07 (-1.0, 0.8)	0.877
Triglyceride, mmol/L ^b^	0.8 (0.4)	0.9 (0.4)	0.04 (-0.1, 0.1)	0.430
HDL-Cholesterol, mmol/L ^b^	1.6 (0.4)	1.6 (0.4)	0.01 (-0.1, 0.1)	0.865
Plasma glucose, mmol/L ^b^	4.8 (0.3)	4.9 (0.3)	0.08 (-0.1, 0.2)	0.206
Insulin, pmol ^b^	41.0 (20.6)	49.0 (23.6)	7.9 (-1.6, 17.4)	0.098
*C-reactive protein*, mg/L ^b,c^	0.7 (0.5, 2.0)	0.7 (0.4, 1.2)	-0.11 (-0.6, 0.4)	0.648
**Work-related outcomes**				
*Work performance (1-10)*	7.1 (0.7)	7.3 (0.7)	0.2 (-0.2, 0.5)	0.356
*Job satisfaction (1-5)*	4.2 (0.6)	4.4 (0.8)	0.1 (-0.2, 0.5)	0.378

Mean changes assessed via paired t-tests (p<0.05 was considered statistically significant).

Min/8-hr workday = minutes at the workplace, standardized to 8 hours of work time (i.e. standardized min = min * 8/observed hours at the workplace)

^a^ n=20; ^b^n=22

c Log transformed values used in the paired t-test analyses and presented.

Work performance: 9-item scale [27] Higher scores indicates better performance.

Job satisfaction single item question. Higher score indicates higher satisfaction.

There were no statistically significant changes in health-related outcomes pre-post move. However, the 95% CIs for weight (-1.1, 0.5kg), percent fat (-1.0, 0.8%), and insulin (-1.6, 17.4 pmol) do not exclude the possibility of meaningful change. For work-related outcomes, there was a small, but non-significant improvement in work-performance and job satisfaction post-move. All participants agreed/strongly agreed that they enjoyed their new workplace, while 75% agreed/strongly agreed it improved their productivity (n=5 were neutral).

## Discussion

The design and construction of a new, ‘activity-permissive’ building enabled the unique opportunity to evaluate the effect of large-scale changes to the workplace on activity-, health-, and work-related outcomes. Post-move, a statistically significant increase in objectively-measured workplace standing time was observed, with a concurrent (though non-statistically significant) decrease in sitting time. Potentially meaningful effects were observed for percent body fat (beneficial) and insulin (detrimental), while both work performance and job satisfaction improved post-move. Importantly, all participants enjoyed the new workplace, and most reported an increase in self-rated productivity post-move.

Although the average changes observed were not large, there was wide individual variability for the sitting, standing, and stepping changes. The average change in sitting time observed (-20 minutes/8-hr workday or -4.1% change) was very similar to that observed following a pre-post intervention evaluating point-of-choice prompting software for reducing prolonged sitting time (-4.4% reduction in sitting time observed) [2], though substantially less than has been observed when all participants receive an individual-level physical environment modification (sit-stand workstation) [4,22]. Differences were observed by employment level, with staff having greater reductions in sitting time, and greater increases in standing time compared to faculty. This suggests that access to the flexible desks were not the primary driver for the change.

It is important to note that participants were not provided with information or education regarding the benefits of reducing prolonged workplace sitting and/or how the new building could help them achieve this. Thus, the change observed could be considered to be primarily reflective of these large-scale environmental changes. Effects may be more substantial if environmental change occurs in combination with interventions based on individual (e.g., goal setting), social (e.g., addressing cultural norms around sitting and moving at the workplace), and organizational-level (e.g., visible organizational support; policies to support standing and moving) change strategies. Such multicomponent interventions, with rigorous evaluations, are needed [4,6,19].

Interestingly, given that the new building was designed to be ‘activity-permissive’, there were minimal changes in stepping time pre-post move. This lack of significant or meaningful change in workplace stepping time is consistent with a previous workplace intervention in office workers that used a multi-component approach (incorporating individual, organizational, and individual-environmental strategies) to encourage office workers to “stand up, sit less, move more” [4]. However, this measure (workplace stepping time) evaluates just one element of physical activity. Collection of data in future research on the type of physical activity (e.g., were there changes in stair use?), and also whether activity outside of the workplace changed (i.e., did the new facilities support more active travel?) will further inform the activity benefits of the physical building design.

There are important implications of these preliminary findings for workplace health promotion, and public health more broadly [18]. Although large-scale modifications to the physical environment are expensive, once built, the intervention (i.e. the structural changes of the building) is on going, and any potential benefits impact all employees (i.e., not just research participants). Furthermore, this study suggests that such benefits may not just be limited to change in activity, but also to work-related outcomes.

Strengths of this study include the objective measurement of both activity and cardio-metabolic outcomes. However, although all employees moving to the new facility were invited to participate, the moderate (34%) response rate meant that the study was underpowered: particularly for changes in health- and work-related outcomes [4]. Changes in activity outside of the workplace were not examined. Furthermore, the sample consisted primarily of highly educated researchers and trained staff working at an academic physical activity research centre. Nevertheless, the proportion of time at the workplace spent sitting (76%, with 55% of this time accrued in prolonged bouts of at least 30 minutes) and stepping for this group was similar to that observed in more general office-based populations [1,4]. Transition to the new building was not something that could be randomized, thus the study design was a natural experiment (i.e., pre-post observational investigation without a control group for comparison). Although this design allows for the observation of workers in real world conditions, it does limit inferences regarding causality, and confounding remains a possibility - particularly given the different seasons for data collection [29].

## Conclusion

This preliminary investigation provides novel objective evidence that the broader physical workplace environment can impact on workplace physical activity, with a move to an “activity-permissive” building resulting in increased workplace standing time. Further research is required to understand the long-term health and work-related impacts of this change, and to evaluate whether there are any further benefits associated with addressing the individual, social and organizational factors that impact on workplace activity across the spectrum, from sitting time through to time spent in moderate- and vigorous-intensity activity.
